# Graviperception in maize plants: is amyloplast sedimentation a red herring?

**DOI:** 10.1007/s00709-018-1272-7

**Published:** 2018-06-11

**Authors:** Hans Georg Edelmann

**Affiliations:** Universitat zu Koeln, Biologiedidaktik, Cologne, Germany

**Keywords:** Graviperception, Gravitropism, Gravi-model, Plant signal transduction, Gravitropic growth, Starch-statolith hypothesis, Tensegrity model, Ethylene

## Abstract

Land plants perceive gravity and respond to it in an organ-specific way; shoots typically direct growth upwards, roots typically downwards. Historically, at least with respect to maize plants, this phenomenon is attributed to three sequential processes, namely graviperception, the transduction of the perceived signal, and the graviresponse, resulting in a typical (re)positioning of the organ or entire plant body relative to the gravivector. For decades, sedimentation of starch-containing plastids within the cells of special tissues has been regarded as the primary and initiating process fundamental for gravitropic growth (starch-statolith hypothesis). Based on Popper’s falsification principle, uncompromising experiments were executed. The results indicate that the model of graviperception based on amyloplast sedimentation does not apply to maize seedlings.

## Introduction

The successful development of a land plant depends from the very beginning on the right direction of growth and positioning of the seedling organs relative to the gravivector. This capacity ensures the plant anchors its roots in the soil (providing nutrients and water) and adjusts light-harvesting shoot organs optimally above ground (enabling photosynthesis).

Growth dependence on gravity—so-called gravitropic growth—is from its early investigations formally divided into three sequential processes: graviperception, the transduction of the perceived stimulus, and the growth response, adjusting the growth and the position of cells or organs according to prevailing graviconditions (Kutschera 2001). The way plants supposedly perceive gravity stems from a long-standing model which arose from the early discoveries of the function of statocyts in animals, when Noll (1892) suggested that plant cells might contain microscopic particles which may function in the same way as animal statoliths. Soon, it was demonstrated that gravisensitive organs of land plants possessed cells containing starch grains which, unlike normal storage starch, sedimented to the cell bottom whatever the position of the organ (Haberlandt 1900; Nemec 1900; Darwin 1903; Rawitscher 1932). From then on, sedimentation of these dense structures within specialized cells (statocytes) of certain tissues has been postulated as the graviperceiving step, supposedly providing the necessary information to optimally position the organs relative to gravity by appropriate growth (responses) and by this to ensure the survival of the individual plant (Wilkins 1966).

In roots, statolith-containing statocytes are ascribed to the columella cells at the very tip of the roots—the so-called root cap—whereas in shoots, cells containing sedimentable amyloplasts are restricted to the so-called starch sheath surrounding the vascular bundles (Noll 1892).

This tissue-restricted and organ-specific model of plant graviperception has since been repeatedly substantiated in a great many of apparent, yet indirect experiments. Both, occurrence of amyloplasts and their sedimentation developed a dogmatic character, disregarding models not involving this cybernetic principle. Yet, how sedimentation yields positional information and how this may eventually induce a hypothetical, factually unknown signal transduction is still, after more than 100 years, speculative. Various modifications of the classical hypothesis of the graviperceiving process are typified in the topographic model, the kinetic model, and the deformation model, and many other versions, all of which aim to rationalize the existence and dependence of these plastids on gravity. In support of such a sedimentation scenario, the cytoskeleton of the cells was claimed to play a crucial role leading to the so-called tensegrity model (Yoder et al. 2001; Ingber 2003), which was suggested to interfere with mechanosensitive ion channels within the cell plasma membrane of plant cells (Yoder et al. 2001). Contradicting this, Yamamoto and Kiss (2002) suggested actin filaments were not involved in gravitropism of stems and hypocotyls. Also, molecular genetic approaches employing knockouts of actin showed no effect on gravitropism (Gilliland et al. 2003) despite demonstrated impacts on auxin transport and altered cycling of PIN1, an auxin transport protein (Muday and Murphy 2002). Although it has also been repeatedly demonstrated that starchless *Arabidopsis* mutants respond gravitropically and therefore do not depend on starch-containing amyloplast sedimentation for graviperception (Caspar and Pickard 1989), the long-standing model developed a dogma-like character, excluding and preventing any models not involving amyloplast sedimentation in specialized cells for graviperception.

As briefly outlined, graviperception via organ-specific plastid sedimentation was always considered “compulsory” for the rationale of any graviperception model. Yet, despite its putatively obvious and, therefore, tempting plausibility, no binding causal processes, i.e., steps causally being induced by sedimentation and themselves inducing processes eventually leading to differential growth, have so far been demonstrated. After more than 100 years, no answers exist to questions such as “what processes represent the signal transduction steps as induced by sedimentation eventually leading to gravitropic differential growth?” or “what signal(s) pass from the site of perception to the site of action?” (Cleland 1997) and “how are sedimenting starch particles related to PIN/auxin redistribution supposedly accomplishing unequal growth of opposing organ flanks?” In fact, despite apparently overwhelming evidence consistently reiterated in favor of the outlined model, we do not know what sedimentation actually does — yet, it is dictating experimental approaches and excluding alternative scenarios. The possible fatal consequences of such a strategy have already been pointed out by August Weismann (1868) saying “although a scientific hypothesis can never be proved, it can be refuted if false, and it therefore raises the question whether facts cannot be taught which are inextricably contradictory with one of the two hypotheses and thus bring it to a collapse....”

In view of the delineated situation, a stringent and clear-cut approach for clarification of this long-standing issue appeared in the concrete and complete surgical elimination of the presumed graviperceiving tissues and the study of the organ behavior without these structures. On the basis of Popper’s falsification approach, or critical empirism (Chalmers 1999), the following hypotheses were formulated:

- If sedimentation of statoliths within the statocytes of the root cap represents the graviperceiving step of gravitropic growth regulation in roots, then its removal should eliminate gravitropic growth.
- If sedimentation of statoliths within the vascular bundle sheath cells represents the graviperceiving step of gravitropic growth regulation in shoots, then their removal should eliminate gravitropic growth.

The system of choice is the classical system for the analyses of graviperception, namely coleoptiles of maize (Wolverton et al. 2002), as well as roots of maize, characterized by so-called closed root caps (Barlow 2002). Both systems allowed surgical removal without destruction, damage, or impairment of the responding tissues.

## Materials and methods

Maize kernels (Hybridmais, Ronaldinio, KWS) were germinated in darkness at room temperature (~ 21–24 °C) by rolling them in moistened sheets of filter paper (MN 710; 580 × 580 mm). For this, 20 kernels were placed in rows at interval distances of 1–1.5 cm on chromatography paper sheets (40 × 10 cm). The rolled sheets were placed vertically in 200-mL glass beakers and filled with distilled water to a depth of 1 cm. The beakers were then covered with aluminum foil. After 2–3 days, the germinated seedlings with developed coleoptiles and also exhibiting roots with lengths in the range of 2 to 3 cm were selected for the experiments (for details, also see Hahn et al. 2006).

Coleoptiles, 2–3 cm in length, were harvested in dim white light. Segments, 2.5 cm in length including the tips, were cut and the primary leaves removed. Coleoptiles were placed in holes in Perspex blocks and stabilized with “plastic-fermit” (Installationskitt; Nissen & Volk, Hamburg, Germany). The blocks were placed in Perspex containers so that the coleoptiles were in a horizontal position. The containers were filled with 350 mL of distilled water, sufficient to cover the coleoptiles, and aerated.

**Fig. 1:**
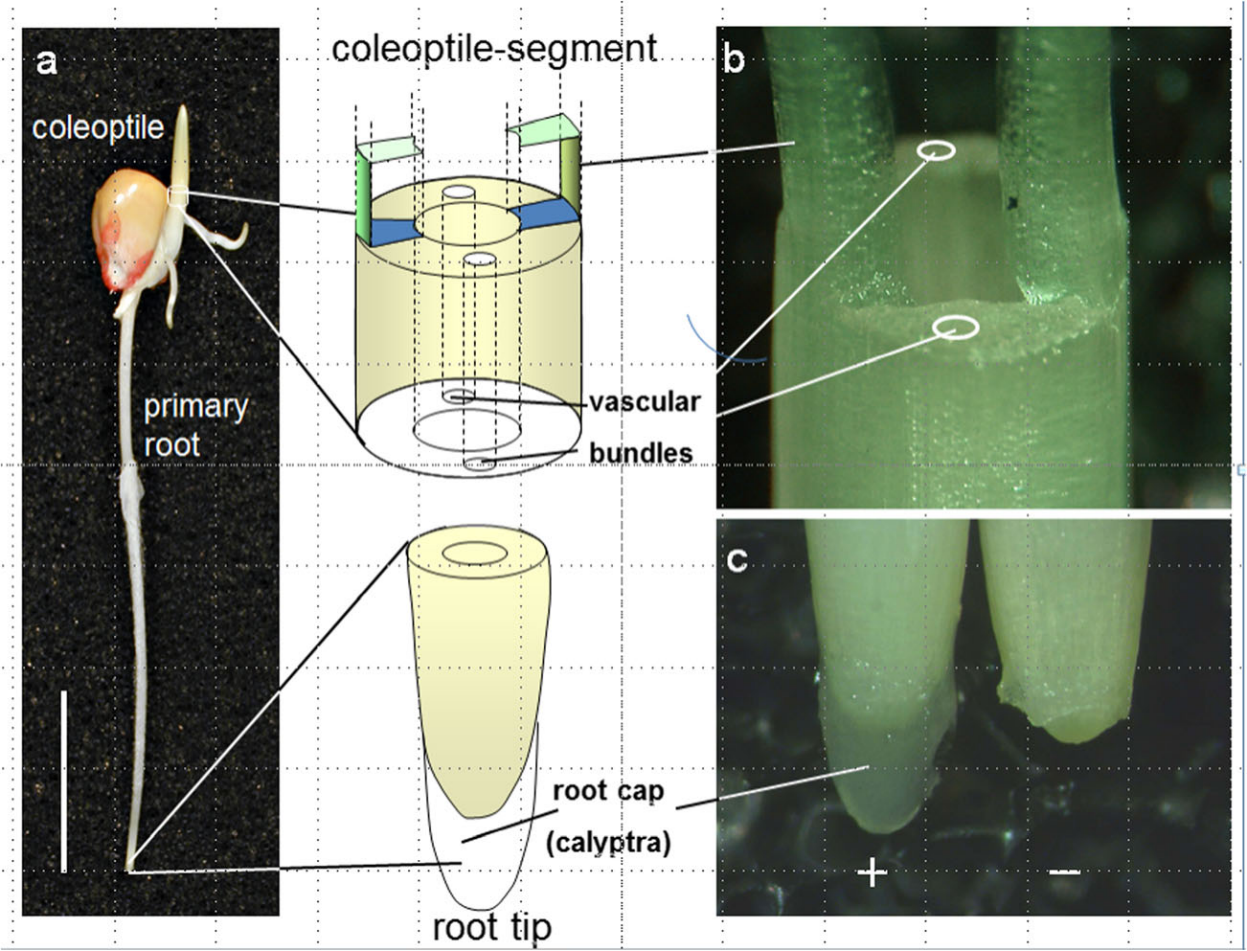
**a** Typical 3-day-old maize seedling as germinated in the dark at room temperature, exhibiting the coleoptile, kernel, and the primary root (vertical bar represents 1-cm length). **b** Coleoptile basis after removal of the enclosed primary leaf and surgical removal of the vascular bundle-containing tissues of the coleoptile illustrating the basis of the remaining longitudinal tissue arches without vascular bundle tissue. **c** Root tip with (+) and without (−) root cap

Removal of the vascular bundles and the surrounding amyloplast-containing tissues of the coleoptiles was carried out by two parallel longitudinal cuts along the coleoptile and two rectangular at the coleoptile basis resulting in ogive-shaped structures. By this, a slim vascular bundle-free tissue kept the two residual coleoptile strips at the very tip together. As referred to cross-sectional tissue area, 80 to 90% of tissue were removed by this step. Similar to intact coleoptiles, adequately prepared coleoptiles were then placed in incubation solutions by fixing their residual basis in well-fitting holes of Perspex blocks (Fig. 1).

Decalyptration of the roots was carried out by a faint partial lateral cut in the zone between the root tip and the beginning of the root cap by pressing the root gently under a binocular on to a vertically fixed razor blade without cutting but causing a predetermined breaking crack. Thereafter, the root cap was carefully removed by squeezing it off between the fingernails.

Root gravitropic growth of both, intact and decapped roots by horizontally placing seedlings on water-imbibed filter paper on top of Styrofoam stands within PET containers (“Gerda-Dosen”). The container bottoms were covered with water-imbibed filter paper in order to guarantee water-saturated air conditions. Decapped roots were preincubated with the roots immersed in Eppendorf caps in solutions of 10 μM latrunculin for 1 h before placing them similar to controls.

## Results and discussion

**Fig. 2:**
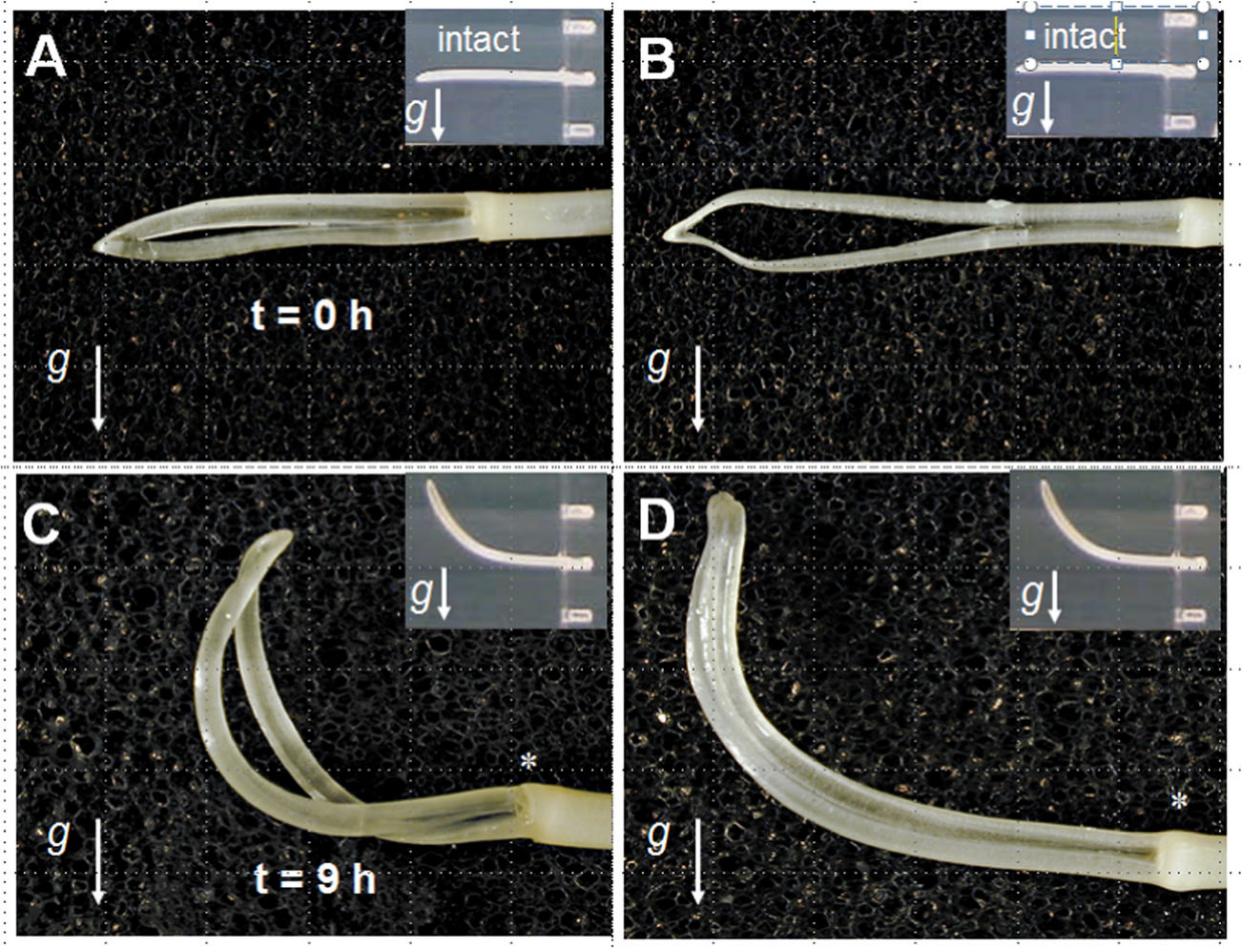
Typical images of **a**, **b** horizontally positioned maize root coleoptiles without vascular bundle sheets at time zero; inserts indicate intact coleoptiles (i.e., containing vascular bundle sheets); **c**, **d** the same coleoptiles after 9 h of gravistimulation (inserts indicate adequately incubated intact coleoptiles). g indicating direction of the gravivector

For coleoptiles, it can be demonstrated that surgical removal of the tissue embedding the vascular bundle with the surrounding amyloplast-containing sheath (see Fig. 1) has no impact on gravitropic growth of horizontally gravistimulated residual coleoptile arches (Fig. 2). In fact, growth response dependence on gravity of the ogive-shaped coleoptile strips, being held together at the very tip by a very thin, maximally 1-mm-broad strip of tissue, is identical to controls, i.e., intact graviresponding coleoptiles (insert, Fig. 2). Apart from the question, whether they would be sufficiently sedimenting, it cannot be ruled out that there might be some starch-filled plastids present in some of the cells of the residual ogive-shaped construct (which in average accounts for 10 to 20% of tissue mass of intact coleoptiles). However, in case these played a causal role in gravity perception, one would expect a drastically inhibited gravitational reaction—which is quite obviously not the case. In addition, single-tissue strips, free of sedimentable amyloplasts, of about 1 cm in length and 2 mm wide horizontally spiked in aerated water on thin needles showed gravitropic elongation behavior in dependence on their positioning as lower or upper flank (data not shown).

After 9 h of incubation, both, intact and coleoptiles without amyloplast-containing tissues exhibit identical graviresponses, which tended to be, but—but not statistically significant — slightly earlier in coleoptiles lacking the vascular bundle sheath tissue established compared to intact coleoptiles, i.e., faster, which might be interpreted by more favorable mechanical conditions for bending.

**Fig. 3:**
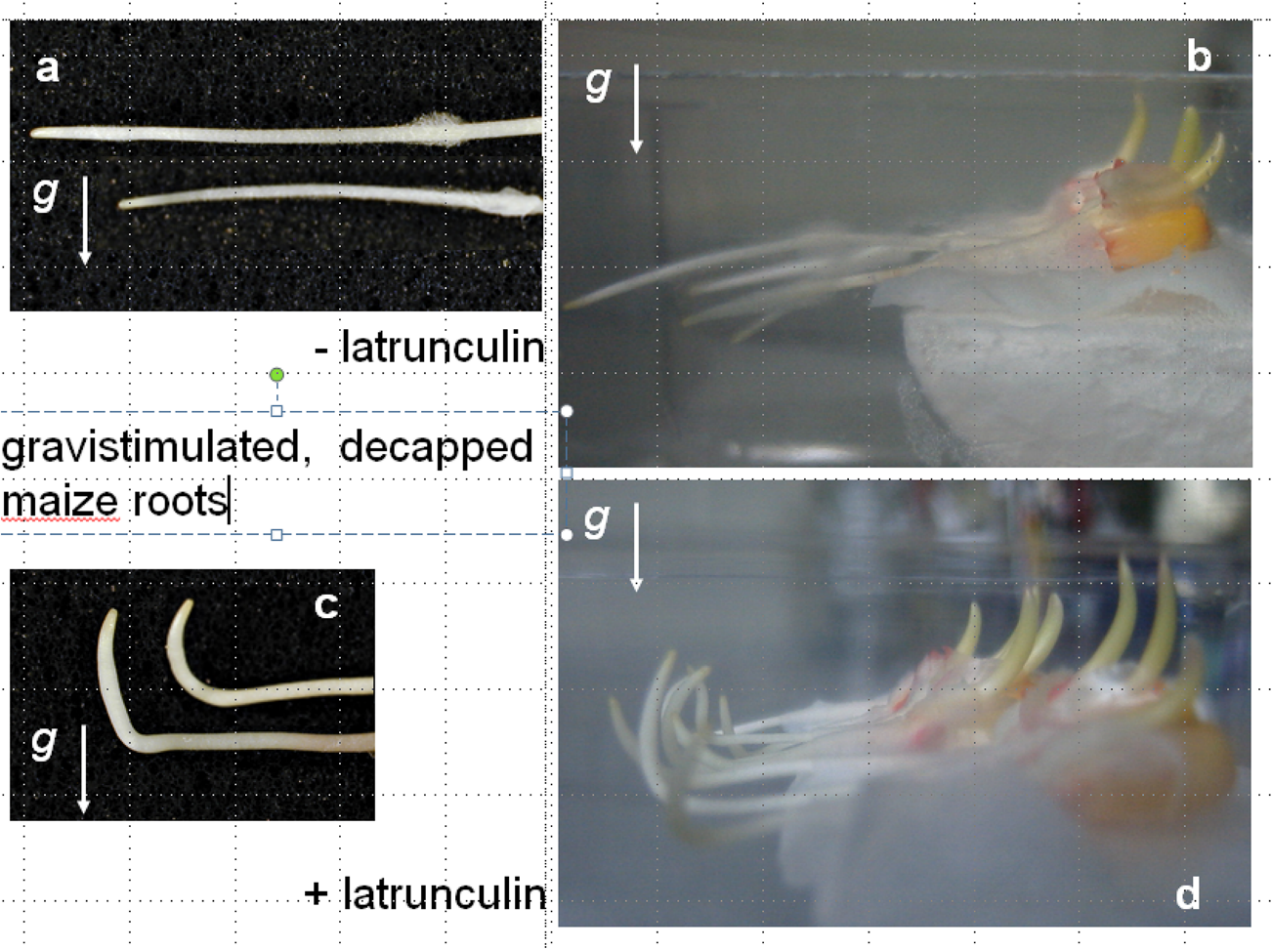
**a**, **b** Different magnification images of decapped roots of maize seedlings horizontally gravistimulated for 24 h. **c**, **d** Root graviresponse of decapped roots incubated for 1 h in latrunculin previous to horizontal gravistimulation

Also in roots (Fig. 1), surgical removal of the generally presumed graviperceiving organ, namely the calyptra with its statocyte-containing columella cells did not result in the effect expected if the statoliths within this tissue represented the graviperceiving organelles. As illustrated in Fig. 3, intact, horizontally gravistimulated roots exhibit a typical growth response which was, similar to previous findings (Hahn et al. 2006), never vertical, but at an angle of about 10° from the vertical. In comparison, decapped roots do not exhibit gravicurvature growth as demonstrated in numerous earlier studies (Hahn et al. 2008). However, this finding does not necessarily imply that the root as such, i.e., without the root cap, does not perceive gravity. It only allows the conclusion that within the intact root, the root cap seems to play an important role for the regulation of gravitropic growth—nothing more and nothing less. In support of this, earlier results imply that the perception step also takes place outside the calyptra (Morita 2010). Such judgment is indicated by the finding that roots briefly incubated for 1 h in a vertical position before gravistimulation in latrunculin, as an inhibitor of actin polymerization (Mancuso et al. 2006), do respond gravitropically without the root cap, yet in a negative manner. Such a growth behavior on dependence on gravity is not observed in water-supplied decapped roots. This pharmacological impact therefore obviously uncovers a capacity endogenous to the root itself. In previous studies on the effect of latrunculin on root growth (Hou et al. 2003, Mancuso et al. 2006), similar effects were reported for decapped, however, positively graviresponding, roots. Furthermore, these authors also demonstrated faint, yet significant gravitropic growth of decapped, water-incubated, i.e., untreated maize roots, indicating a potential for gravitropic growth, which seems to be restrained without the cap somehow and which is relieved under the influence of latrunculin.

As illustrated in Figs. 2 and 3, both organs lacking the presumed graviperceiving tissues exhibit graviresponsive growth. Sedimenting, starch-filled amyloplasts are quite obviously not necessary for growth dependent on gravity.

Based on these clear and unequivocal findings, one may ask: what other or further removal of a claimed graviperceiving system has to be carried out in order to demonstrate its absence as irrelevant for gravitropic growth? Or—vice versa—“why should we attribute to a tissue or an organ a regulatory function when its elimination does not result in a deletion or strongly impeded graviresponse?”

Consistent with earlier presented data (Firn and Digby 1977) in which the loss of gravitropic growth was demonstrated by removal of the epidermal tissues, the finding implies that not only accomplishment of gravibending, but also anticipatory perception of gravity takes place within the peripheral tissue—without sedimentation of amyloplasts. In support of this, Wolverton et al. (2002) demonstrated the existence of root-borne graviperception, yet without any idea on “the nature of the signal originating outside the root cap.”

It therefore appears appropriate to search for alternative mechanisms for graviperception (also) immanent to peripheral cells, irrespective of the lack of sedimentable amyloplasts.

There are apprehensible explanations for the rationale of the ancient model of graviperception. By then, in the absence of any other measurable effect accompanying reorientation of a plant organ, the obvious (re)sedimentation in dependence of the cells position relative to the gravivector provided a most plausible scenario as the graviperceiving step. However, as in context with many other scientific problems, plausibility can be a tempting, yet a “dangerous teacher.”

In fact, the model of graviperception or gravitropic growth regulation in general might look very different had scientists in the early days of exploring gravitropism known about and had the chance to detect and to measure ethylene. It has been reported that inhibiting ethylene synthesis results in a concentration-dependent manner, in a corresponding reduction of gravitropic growth of (Edelmann and Roth 2006). The more ethylene synthesis inhibited, the less pronounced gravicurvature. How these ethylene-dependent phenomena are brought about is still speculative. Nevertheless, it may be worthwhile to address ethylene as a serious candidate for graviperception.

In support of this, according to results of more recent studies (reviewed by Sato et al. 2015), models of gravitropic growth regulation based on (re)sedimentation of amyloplasts in columella cells are redundant: PIN—proteins facilitating gravi-dependent auxin transport—redistribute per se dependent on gravity (Gälweiler et al. 1998; Müller et al. 1998; Friml et al. 2002). This effect does not depend on sedimentation of amyloplasts, the (ir)relevance of which seems deliberately not addressed—at least not in cybernetical detail—since it is obviously not needed for the gravitropic scenario.

## References

[CR1] Barlow PW (2002). The root cap: cell dynamics, cell differentiation and cap function. J Plant Growth Regul.

[CR2] Caspar T, Pickard BG (1989). Gravitropism in a starchless mutant of Arabidopsis: implications for the starch-statolith theory of gravity sensing. Planta.

[CR3] Chalmers AF (1999). What is this thing called science?.

[CR4] Cleland RE (1997). General discussion on graviresponses. Planta.

[CR5] Darwin F (1903). The statolith theory of geotropism. Nature.

[CR6] Edelmann HG, Roth U (2006). Gravitropic plant growth regulation and ethylene: an unsought cardinal coordinate for a disused model. Protoplasma.

[CR7] Firn R, Digby J (1977) The role of the peripheral cell layers in the geotropic curvature of sunflower hypocotyls: a new model of shoot geotropism. Funct Plant Biol:1–11

[CR8] Friml J, Wisniewska J, Benkova E, Mendgen K, Palme K (2002). Lateral relocation of auxin efflux regulator PIN3 mediates tropism in Arabidopsis. Nature.

[CR9] Gälweiler L, Guan C, Müller A, Wisman E, Mendgen K, Yephremov A, Palme K (1998). Regulation of polar auxin transport by AtPIN1 in *Arabidopsis* vascular tissue. Science.

[CR10] Gilliland LU, Pawloski LC, Kandasamy MK, Meagher RB (2003). Arabidopsis actin gene *ACT7* plays an essential role in germination and root growth. Plant J.

[CR11] Haberlandt G (1900). Ueber die Perception des geotropischen Reizes. Ber Deut Bot Ges.

[CR12] Hahn A, Firn R, Edelmann HG (2006). Interacting signal transduction chains in gravity-stimulated maize roots. Signal Transduction.

[CR13] Hahn A, Zimmermann R, Wanke D, Harter K, Edelmann HG (2008). The root cap determines ethylene-dependent growth and development in maize roots. Mol Plant.

[CR14] Hou G, Mohamalawari DR, Blancaflor EB (2003). Enhanced gravitropism of roots with a disrupted cap actin cytoskeleton1. Plant Physiol.

[CR15] Ingber DE (2003). Tensegrity I. Cell structure and hierarchical systems biology. J Cell Sci.

[CR16] Kutschera U (2001). Gravitropism of axial organs in multicellular plants. Adv Space Res.

[CR17] Mancuso S, Barlow PW, Volkmann D, Baluska F (2006). Actin turnover-mediated gravity response in maize root apices gravitropism of decapped roots implicates gravisensing outside of the root cap. Plant Signal Behav.

[CR18] Morita MT (2010). Directional gravity sensing in gravitropism. Annu Rev Plant Biol.

[CR19] Muday GK, Murphy AS (2002). An emerging model of auxin transport regulation. Plant Cell.

[CR20] Müller A, Guan C, Gälweiler L, Tänzler P, Huijser P, Marchant A, Parry G, Bennett M, Wisman E, Palme K (1998). AtPIN2 defines a locus of Arabidopsis for root gravitropism control. EMBO J.

[CR21] Nemec B (1900). Über die Art der Wahrnehmung des Schwerkraftreizes bei den Pflanzen. Ber Deut Bot Ges.

[CR22] Noll F (1892). Über heterogene Induktion: Versuch eines Beitrags zur Kenntnis der Reizerscheinungen der Pflanzen.

[CR23] Rawitscher F (1932). Der Geotropismus der Pflanzen. 420 S.

[CR24] Sato EM, Hussein Hijazi H, Bennett MB, Vissenberg K, Swarup R (2015). New insights into root gravitropic signalling. J Exp Bot.

[CR25] Weismann A (1868). Über die Berechtigung der Darwin’schen Theorie. Leipzig.

[CR26] Wilkins MB (1966). Geotropism. Annu Rev Plant Physiol.

[CR27] Wolverton C, Ishikawa H, Evans ML (2002). The kinetics of root gravitropism: dual motors and sensors. J Plant Growth Regul.

[CR28] Yamamoto K, Kiss JZ (2002). Disruption of the actin cytoskeleton results in the promotion of gravitropism in inflorescence stems and hypocotyls of Arabidopsis. Plant Physiol.

[CR29] Yoder TL, Zheng HQ, Todd P, Staehelin LA (2001). Amyloplast sedimentation dynamics in maize columella cells support a new model for the gravity-sensing apparatus of roots. Plant Physiol.

